# The Effect of Hormonal Contraception Use on Ovarian Reserve Markers and the Uptake of Assisted Reproductive Technology in Individuals Seeking an Infertility Evaluation

**DOI:** 10.7759/cureus.40927

**Published:** 2023-06-25

**Authors:** Dana R Siegel, Joellen Fresia, Angela Fought, Jeanelle Sheeder, Karen Hampanda, Leslie Appiah

**Affiliations:** 1 Obstetrics and Gynecology, University of Colorado School of Medicine, Aurora, USA; 2 Biostatistics and Informatics, University of Colorado School of Medicine, Aurora, USA; 3 Obstetrics and Gynecology/Clinical Research, University of Colorado School of Medicine, Aurora, USA; 4 Center for Global Health, Obstetrics and Gynecology, University of Colorado School of Medicine, Aurora, USA

**Keywords:** assisted reproductive technology (art), live birth rate, anti-müllerian hormone, ovarian reserve, hormonal contraception

## Abstract

Background and objective

The effects of hormonal contraception (HC) use on ovarian reserve (OR) markers in individuals seeking an infertility evaluation and the success of assisted reproductive technology (ART) warrant further investigation. Therefore, the aim of this study was to determine if women seeking an evaluation for unexplained infertility who used long-term (≥2 years) HC have lower ovarian reserve (OR) markers and higher uptake of ART compared to short-term (<2 years) or never HC users.

Methods

We performed a cross-sectional patient survey involving a retrospective medical chart review of patients seeking an evaluation for unexplained infertility at the University of Colorado Advanced Reproductive Medicine (CU ARM) clinic.

Results

Most participants (87%; 107/123) reported a history of HC use with 98 (79.7%) reporting long-term continuous use for two or more years. Median OR markers were similar between long-term and short-term/never HC users [anti-Müllerian hormone (AMH): 2.4 vs. 3.2, p=0.20; antral follicle count (AFC): 18 vs. 26, p=0.10; follicle-stimulating hormone (FSH): 7.6 vs. 6.3, p=0.26] and remained so after adjusting for age and diagnosis of polycystic ovarian syndrome (PCOS) or primary ovarian insufficiency (POI) in linear regression models. However, among HC users aged less than 30 years (n=9), those who had discontinued HC between two and three years prior to the assessment of their OR markers had a 6.20 ng/mL increase in AMH level compared to those who had discontinued HC less than two years prior to the assessment (p=0.02). Additionally, there was a marginally increased use of ART overall among long-term HC users compared to short-term/never HC users (64.3% vs. 44.0%, p=0.06), specifically in the use of in vitro fertilization (IVF) (58.7% vs. 18.2%, p=0.01). Among long-term HC users, ovulation induction was less likely to result in live birth compared to short-term/never HC users (8.9% vs. 62.5%, p<0.001); however, after adjusting for age, PCOS, POI, and type of ART used, there was no difference in the odds of live birth after ART between long-term HC users and short-term/never users.

Conclusion

While long-term HC users report increased use of ART, in particular IVF, the overall conception rates and live birth outcomes among ART users do not appear to be significantly affected by a history of long-term HC use.

## Introduction

According to the National Survey of Family Growth, approximately 65% of women of reproductive age are currently using some form of contraception [1]. Yet, a recent meta-analysis found that women frequently report concerns about the potential side effects related to infertility when asked about contraceptive initiation, causing hesitation among them to start or continue the use of contraception [2]. In the United States, there is an increasing prevalence of women of reproductive age opting to use hormonal contraception (HC) rather than non-hormonal options, for various reasons [3]. The most frequent reason for the initiation of HC is to prevent or delay pregnancy; women also request HC to reduce or eliminate monthly uterine bleeding, prevent or lessen acne, and manage menstrual cramps, among other reasons [4-6]. Current HC options include combined oral contraceptive pills (COCPs), transdermal patches, vaginal rings, progestin-only contraceptive pills (POPs), injectables, implants, and levonorgestrel-releasing intra-uterine devices (LNG-IUDs). Although the precise mechanism of action of each of these HC methods varies, to some degree, they all influence and prevent ovulation either fully or partially by suppressing the hypothalamic-pituitary-ovarian (HPO) axis [7]. Prior studies have found conflicting results regarding the effects of this suppression on ovarian reserve (OR) markers [8-15]. In light of this, there is a need to further explore the lasting effects of long-standing HC use on OR and on the use of artificial reproductive technology (ART) and fertility outcomes.

As people continue to delay childbearing for various reasons, OR assessment has become an important tool to estimate one’s remaining reproductive lifespan as well as to predict the success rate among those undergoing treatment for infertility including ART. Currently, the most commonly used OR markers include anti-Müllerian hormone (AMH), follicle-stimulating hormone (FSH), and antral follicle count (AFC). Of these, AFC is the most reliable direct marker of OR. However, utilization of AFC is limited by the need for a skilled ultrasonographer and the invasive nature of the transvaginal ultrasound required for accurate evaluation. Given these limitations, serum AMH is considered to be the superior parameter of age-related ovarian follicle depletion and thus reproductive capacity, particularly in those undergoing controlled ovarian stimulation during ART [16]. AMH is a glycoprotein released from the granulosa cells of large pre-antral and small antral follicles and the serum hormone levels have been shown to correlate with the number of developing primordial follicles [17].

However, various studies have shown that pharmacological interventions including HC may affect AMH levels, thereby making the utility of this marker difficult to interpret in HC users. For example, a study by Bentzen et al. showed that serum AMH, AFC, and ovarian volume were all lower among HC users and this was negatively associated with the duration of contraception use [14]. Various other studies have corroborated these findings, showing a reduction in AMH levels of between 19%-31% among HC users [12,13,15,18,19] as well as reductions in AFC and ovarian volume [9,20]. However, contradictory findings have been reported by Deb et al. and several others who found a non-significant difference in serum AMH levels between HC users and non-users [8-11,21,22]. Other studies indicate that the effect of HC use on OR markers is likely temporary as AMH levels tend to normalize after the cessation of HC use [11] and should not be correlated with the cumulative duration of use [19].

In addition to the mixed results on the effects of HC on OR markers, the clinical significance of such alterations on one’s fertility potential, the subsequent prevalence of evaluation by Reproductive Endocrinology and Infertility (REI) specialists, and the utilization and outcomes of ART have not been well-studied. Among patients without a diagnosis of infertility, Berglund Scherwitzl et al. found that previous HC users have, on average, a slightly longer time to pregnancy initially (146 days vs. 85 days) but have no differences in pregnancy rates in the long term [23]. Likewise, a comprehensive meta-analysis found similar one-year pregnancy rates among those who had previously used HC including OCPs, implants, injectables, LNG-IUDs and those who had used barrier methods or no contraception at all [24]. Significantly, however, these studies were not limited to an infertility patient population requiring treatment for their family-building goals.

As an increasing number of women are relying on HC with a concurrent increase in the number of women seeking treatment for infertility [25], it is important to look into any long-term effects that HC may have on a woman’s future fertility potential and the biomarkers used to guide infertility diagnosis, along with the success of infertility treatment. Therefore, the aim of this study was to examine if there is an association between the use of long-standing HC (≥2 years) and (1) OR markers, (2) the use of ART, and (3) outcomes of ART among women of reproductive age seeking an evaluation for infertility when compared to short-term (<2 years) or never users of HC. We believe the findings from this study will help inform the current discrepant body of literature on the potential effects of long-standing HC on OR markers as well as highlight the impact on ART use, thereby enabling providers to more appropriately counsel patients seeking family planning evaluation or infertility treatment.

## Materials and methods

Study design

This study used an online-administered patient survey that was linked to a retrospective medical chart review to collect data from women of reproductive age seen at the University of Colorado Advanced Reproductive Medicine (CU ARM) clinic from December 2019 to December 2020. We first created a dataset of women aged between 18 and 45 years who were seen at CU ARM for an infertility evaluation with a primary ICD-10 code of female infertility or female infertility unspecified. Those with ICD-10 codes of pelvic inflammatory disease (PID), Müllerian anomaly, salpingitis, malignancy, and/or male infertility were excluded. The e-mail addresses of those who met our initial inclusion criteria were collected from their medical records and stored within a secure REDCap (Research Electronic Data Capture) database [26]. A REDCap survey questionnaire was developed by the experts in the research team and disseminated to eligible participants via e-mail. To proceed with the survey and enrollment into the study, informed consent was sought from the participants and they were asked to answer screening questions (see Appendix) to check their eligibility for inclusion (aged between 18 and 45 years, never diagnosed with a Müllerian anomaly or PID, never undergone surgery to remove one or both their ovaries or fallopian tubes, and never undergone chemotherapy or radiation). The survey collected data on participant demographics, medical history, menstrual and contraceptive history, reproductive history, use of ART, and outcomes of ART. The participants were within their rights to terminate their participation in the study at any point or skip questions they preferred not to answer. The study was approved by the Colorado Multiple Institutional Review Board (COMIRB).

After obtaining consent and completing the survey, a retrospective chart review was performed and linked to the survey responses. The electronic medical record (EMR) was reviewed to gather the following data on each participant: serum AMH level(s) in relation to time since the discontinuation of HC (if applicable), AFC (highest level if multiple), serum FSH (highest level if multiple), presence or absence of FMR1 mutation, thyroid-stimulating hormone (TSH) level as well as free T4/T3 and the presence or absence of thyroid antibodies, prolactin (PRL) levels, and the most recent hemoglobin A1C test results. The OR markers collected at CU ARM are all examined in the same fashion: AMH levels are analyzed by the Associated Regional and University Pathologists, Inc. (ARUP) Laboratories (Salt Lake City, UT), FSH levels are analyzed by the University of Colorado Health Laboratory at the Anschutz Medical Campus, and AFC measurements are taken by the same set of ultrasonographers. All data were stored within a secure REDCap database.

Study instrument

The data collection tool used in this study was a researcher-created questionnaire comprising 29 items. In addition to the screening questions mentioned above, the questionnaire addressed topics related to the participant’s contraceptive, reproductive, surgical, and medical history. Regarding contraception history, the participant was asked to quantify the longest period of continuous use of any form of HC including COCPs, contraceptive transdermal patches, contraceptive vaginal rings, POPs, contraceptive injectables, implants, and/or LNG-IUDs as well as the time since the discontinuation of aforementioned HC, if applicable. In terms of reproductive history, the questionnaire also inquired whether the participant had ever been pregnant, the outcome(s) of the pregnancy, whether they had used ART, and the type, frequency, and outcome(s) of ART, if applicable. The questionnaire also included four demographic questions, pertaining to age, self-identified race and ethnicity, and marital status.

Statistical analysis

We computed descriptive statistics using IBM SPSS Statistics version 28 (IBM Corp., Armonk, NY), including tests of normality for the overall sample and that stratified by long-term and short-term/never HC users. Participants who reported a continuous use of HC for ≥2 years were categorized as “long-term” HC users and those who had never used HC or had used for <2 years were analyzed together as “short-term/never users”. Proportions were used to describe binary variables. Mean and standard deviation (SD) were used to describe parametric continuous variables. Median and interquartile range (IQR) were used to describe non-parametric continuous variables. We used appropriate bivariate tests (chi-squared test, t-test, or Mann-Whitney Wilcoxon test) to compare characteristics and outcomes between long-term and short-term/never users of HC. 

We created simple and multivariable linear regression models to identify predictors of OR markers (continuous variables) and simple and multivariable logistic regression models to identify predictors of fertility outcomes with ART (binary). Simple and multivariable linear regression models used the OR marker variables of AMH, FSH, and AMC as dependent variables and included the independent variable of interest (long-term vs. short-term/never users of HC and amount of time between discontinuation of HC and OR markers) and were controlled for variables known to be associated with OR markers (age, PCOS, POI). For binary outcomes (pregnancy and live birth after ART), simple and multivariable logistic regression models were used and were controlled for age, PCOS, POI, as well as the type of ART used.

To determine whether age was a moderating factor affecting the relationship between time since discontinuing HC and OR markers, we created a categorical variable (less than 30 years; 30-34 years; 35-39 years; and 40-44 years). Mann-Whitney Wilcoxon tests established significant associations between time since discontinuing HC and OR markers (AMH, FSH, AMC) by age group. Age groups with an association of p<0.1 between time since discontinuing HC and OR markers in the Mann-Whitney Wilcoxon test were tested in simple regression models. Multivariable regression models were not computed due to sub-group age sample sizes being small. All statistics were computed using STATA 16.

## Results

Study population

**Figure 1:**
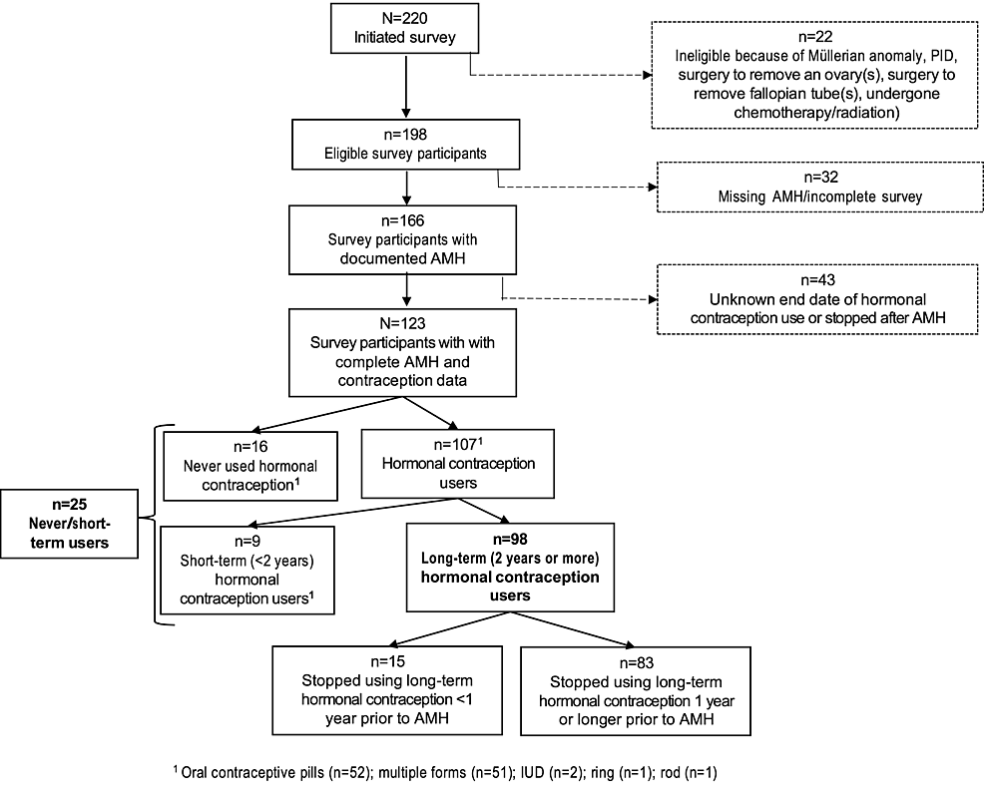
CONSORT diagram depicting the flow of participants CONSORT: Consolidated Standards of Reporting Trials; PID: pelvic inflammatory disease; AMH: anti-Müllerian hormone; IUD: intrauterine device

A total of 1,227 charts were identified and data were extracted based on the above-mentioned inclusion and exclusion criteria; invitations were sent to the women via e-mail seeking their participation in the study. Of these, 31 invitations returned as “undeliverable”, meaning that a total of 1,196 e-mails were successfully sent. A total of 220 participants initiated the survey and 198 of them met the eligibility criteria and completed the survey. Twenty-two patients were deemed ineligible due to one or more of the following exclusion criteria: history of PID (n=1), a Müllerian anomaly (n=4), history of surgery to remove ovary(s) (n=3); history of surgery to remove fallopian tube(s) (n=11); and/or a history of chemotherapy/radiation (n=7). Furthermore, 32 participants were excluded from the analysis because OR markers were unavailable and 43 participants were excluded because they had not discontinued or had an unknown end date for their HC prior to the OR assessment, leaving a final sample size of 123 participants who were included in the primary analysis (Figure 1).

In the sample, 107 participants (87.0%) reported some form of HC use. Use of specific HC methods included COCPs only (n=52); multiple forms of HC (n=51); LNG-IUD only (n=2); ring only (n=1); and rod only (n=1). Ninety-eight participants (79.7%) reported using a form of HC continuously for ≥2 years (“long-term HC users”), while 25 participants (20.3%) were “short-term/never users”, with nine reporting short-term HC use for <2 years and 16 reporting never using HC.

The mean age of the overall sample was 33.4 ± 4.5 years and respondents predominantly self-identified as non-Hispanic Caucasian/White (n=108, 87.8%) and had, on average, been pregnant 1.3 ± 1.5 times. Most participants were married (n=106, 86.2%), and marriage was more commonly reported among long-term HC users compared to short-term/never users (90% vs. 68%, p=0.003). The median body mass index (BMI) was 25.0 kg/m^2^ (overweight) with an IQR of 22.8-30.5. Long-term HC users had a lower median BMI compared to short-term/never users (24.8 kg/m^2^ vs. 28.9 kg/m^2^, p=0.018). Overall, 84% (n=103) of the sample reported difficulty in becoming pregnant with a significantly higher proportion among long-term HC users compared to short-term/never users (87.8% vs. 68.0%, p=0.017). There were no differences in the prevalence of various medical conditions between long-term and short-term/never HC users, including PCOS (19.4% vs. 20.0%, p=0.82), primary ovarian insufficiency (POI) (8.3% vs. 0, p=0.15), thyroid disorders (24.7% vs. 25%, p=0.98), hyperprolactinemia (3.1% vs. 0, p=0.38), or history of a sexually transmitted infection (STI) (19.8% vs. 8.3%, p=0.19). There was only one participant who reported smoking tobacco within the past year (0.9%) (Table 1).

**Table 1:** Participant characteristics stratified by length of continuous hormonal contraceptive use ^1^N=123 women with complete AMH and contraceptive use data. ^2^n=96 stopped using more than 12 months; n=2 stopped less than 12 months prior to AMH. ^3^Non-parametric distribution, Mann-Whitney Wilcoxon test used for comparison. ^4^Among 107 contraception users. ^5^Among 74 participants reporting ART use HC: hormonal contraception; SD: standard deviation; BMI: body mass index; IQR: interquartile range; PCOS: polycystic ovarian syndrome; POI: primary ovarian insufficiency; STI: sexually transmitted infection; AMH: anti-Müllerian hormone; AFC: antral follicle count; FSH: follicle-stimulating hormone; ART: assisted reproductive technology

Variable	Total (N=123)^1^	Long-term HC users (n=98)^2^	Short-term/never HC users (n=25)	P-value
Age, years, mean ± SD	33.4 ± 4.5	33.7 ± 4.1	32.1 ± 5.6	0.10
BMI, kg/m^2^, median (IQR)^3^	25.0 (22.8 – 30.5)	24.8 (22.5 – 29.9)	28.9 (24.3 – 33.2)	0.02
Race, % (n)				0.06
Caucasian/White	87.8% (108)	90.8% (89)	76.0% (19)	
African American/Black	0.8% (1)	1.0% (1)	0	
Asian	6.5% (8)	4.1% (4)	16% (4)	
Multiracial	4.1% (5)	4.1% (4)	4.0% (1)	
Married, % (n)	86.2% (106)	90.8% (89)	68.0% (17)	0.01
Current cigarette use, % (n)	0.9% (1)	1.0% (1)	0	0.61
Diagnoses, % (n)				
Fibroid(s)	10.7% (13)	10.4% (10)	12.0% (3)	0.15
PCOS	19.5% (24)	19.4% (19)	20.0% (5)	0.82
Endometriosis	6.6% (8)	8.3% (8)	0	0.15
POI	6.6% (8)	8.3% (8)	0	0.15
Diabetes	0	0	0	-
Thyroid condition	24.8% (30)	24.7% (24)	25.0% (6)	0.98
Prolactinoma	2.5% (3)	3.1% (3)	0	0.38
STI	17.5% (21)	19.8% (19)	8.3% (2)	0.19
Eating disorder	5.0% (6)	5.2% (5)	4.2% (1)	0.83
Autoimmune disorder	5.0% (6)	5.3% (5)	4.2% (1)	0.83
History of pelvic surgery(s)	35.5% (43)	39.6% (38)	20.0% (5)	0.07
Age of menarche, years, mean ± SD	12.6 ± 1.5	12.5 ± 1.3	13.0 ± 2.2	0.15
Self-reported difficulty in becoming pregnant, % (n)	83.7% (103)	87.8% (86)	68.0% (17)	0.017
Gravida, mean (SD)	1.3 (1.5)	1.2 (1.4)	1.3 (1.6)	0.99
Time since hormonal contraception discontinuation, years^4^, median (IQR)	2.8 (1.3 – 5.6)	2.7 (1.3 – 5.0)	7.1 (4.7 – 13.7)	0.01
Ovarian reserve markers, median (IQR)^3^				
AMH, ng/mL	2.8 (1.2 – 4.7)	2.4 (1.1 – 4.7)	3.2 (2.5 – 3.7)	0.20
AFC, #	21 (13 – 32)	18 (12 – 31)	26 (19 – 35)	0.10
FSH, IU/L	7.2 (5.7 – 9.2)	7.6 (5.7 – 9.3)	6.3 (5.6 – 8.7)	0.26
Ever used ART, % (n)	60.2% (74)	64.3% (63)	44.0% (11)	0.06
Type of ART used^5^, % (n)				
Ovulation induction used, % (n)	71.6% (53)	71.4% (45)	72.7% (8)	0.93
Intrauterine inseminations used, % (n)	66.2% (49)	66.7% (42)	63.6% (7)	0.85
In vitro fertilization used, % (n)	54.7% (39)	58.7% (37)	18.2% (2)	0.01
Surrogacy used, % (n)	1.4% (1)	1.6% (1)	0	0.67
Intra-fallopian transfer used	0	0	0	NA
Number of different forms of ART used, median (IQR)^3, 5^	2.0 (0.0 – 2.0)	2.0 (1.0 – 2.0)	1.0 (0.0 – 2.0)	0.28
Total number of ART attempts, median (IQR)^3, 5^	5.0 (3.0 – 7.0)	5.0 (3.0 – 7.0)	4.0 (1.0 – 7.0)	0.51
Conception with ART, % (n)				
Ever pregnant from any form of ART (n=74)	43.2% (32)	39.7% (25)	63.6% (7)	0.14
Ever pregnant from ovulation induction (n=53)	24.5% (13)	15.6% (7)	75.0% (6)	<0.01
Ever pregnant from Intrauterine insemination (n=49)	26.5% (13)	21.4% (9)	57.1% (4)	0.05
Ever pregnant from in vitro fertilization (n=39)	35.9% (14)	35.1% (13)	50.0% (1)	0.67
Ever pregnant from surrogacy (n=1)	100% (1)	100% (1)	0	NA
Ever pregnant from Intra-fallopian transfer	0	0	0	NA
Live birth with ART, % (n)				
Any form of ART (n=74)	28.4% (21)	23.8% (15)	54.6% (6)	0.04
Ovulation induction (n=53)	17.0% (9)	8.9% (4)	62.5% (5)	<0.01
Intrauterine insemination (n=49)	12.2% (6)	9.5% (4)	28.6% (2)	0.16
In vitro fertilization (n=39)	23.1% (9)	21.6% (8)	50.0% (1)	0.48
Surrogacy (n=1)	0.8% (1)	1.0% (1)	0	0.61
Intra-fallopian transfer	0	0	0	NA

Hormonal contraception use patterns

Among those reporting HC use (n=107), the median number of years since the discontinuation of HC prior to OR evaluation was 2.8 (IQR: 1.3-5.6) (Table 1). The median number of years since the discontinuation of HC in long-term HC users was 2.7 years (IQR: 1.3-5.0), compared to 7.1 years in short-term/never HC users (IQR: 4.7-13.7) (p=0.01). Among the long-term HC users, the most frequently used method was COCPs either alone or in addition to another method (94.9%, n=93), and many had used it for more than 10 years (n=24; 25.8%).

Ovarian reserve markers

Overall, the median serum AMH was 2.8 ng/mL (IQR: 1.2-4.7). Long-term users had a trend towards lower median AMH levels compared to short-term/never users, though this was not statistically significant in the bivariate analysis [2.4 (1.1-4.7) vs. 3.2 (2.5-3.7) ng/mL, p=0.20]. Additionally, there was no significant difference in cycle day three FSH levels between long-term and short-term/never users [7.6 (5.7-9.3) vs. 6.3 (5.6-8.7) IU/L, p=0.26] nor in cycle day three AFC measurements [18 (12-31) vs. 26 (19-35), p=0.10] in bivariate analysis. Lastly, there was similarly no difference in OR markers between long-term and short-term/never users in the simple or adjusted linear regression models after controlling for age and diagnosis of PCOS or POI.

In the simple model, younger age or a diagnosis of PCOS was significantly associated with higher serum AMH level (β = -0.22, p<0.01) (β = 4.81, p<0.01), whereas a diagnosis of POI was significantly associated with a lower AMH level (β = -2.53, p<0.01). In the adjusted linear regression models, younger age was significantly associated with lower cycle day three FSH levels (β = 0.28, p<0.05) and higher day three AFC levels (β = -0.68, p<0.01) whereas a diagnosis of POI was associated with higher day three FSH levels (β = 17.45, p<0.001) and lower day three AFC levels (β = -10.00, p<0.05) (Table 2).

**Table 2:** Simple and multivariable linear regression results for variables associated with ovarian reserve markers *p<0.05. **p<0.01. ***p<0.001 ^1^Reference group: short-term/never HC users. ^2^All variables listed included in the model AMH: anti-Müllerian hormone; AFC: antral follicle count; FSH: follicle-stimulating hormone; PCOS: polycystic ovarian syndrome; POI: primary ovarian insufficiency

Variable	AMH (n=123)		AFC (n=120)		FSH (n=120)	
	Unadjusted model	Adjusted model^2^	Unadjusted model	Adjusted model^2^	Unadjusted model	Adjusted model^2^
	β (95% CI)	β (95% CI)	β (95% CI)	β (95% CI)	β (95% CI)	β (95% CI)
Long-term hormonal contraception use^1^	0.23 (-1.71 – 2.18)	0.68 (-1.07 – 2.43)	-3.54 (-9.06 – 1.78)	-1.96 (-6.69 – 2.77)	1.77 (-1.73 – 5.27)	0.01 (-2.84 – 2.86)
Age, years	-0.22** (-0.39 – -0.06)	-0.15 (-0.31 – 0.00)	-0.91*** (-1.36 – -0.46)	-0.68** (-1.10 – -0.26)	0.37* (0.07 – 0.67)	0.28* (0.03 – 0.53)
PCOS	4.81*** (3.02 – 6.59)	4.31** (2.52 – 6.10)	12.49*** (7.43 – 17.54)	10.43*** (5.60 – 15.26)	-1.85 (-5.34 – 1.65)	0.14 (-2.74 – 3.02)
POI	-3.57* (-6.69 – -0.45)	-2.53 (-5.40 – 0.33)	-14.09** (-23.20 – -4.99)	-10.00* (-18.23 – -1.77)	17.91*** (13.43 – 22.40)	17.45*** (12.92 – 21.98)

**Figure 2:**
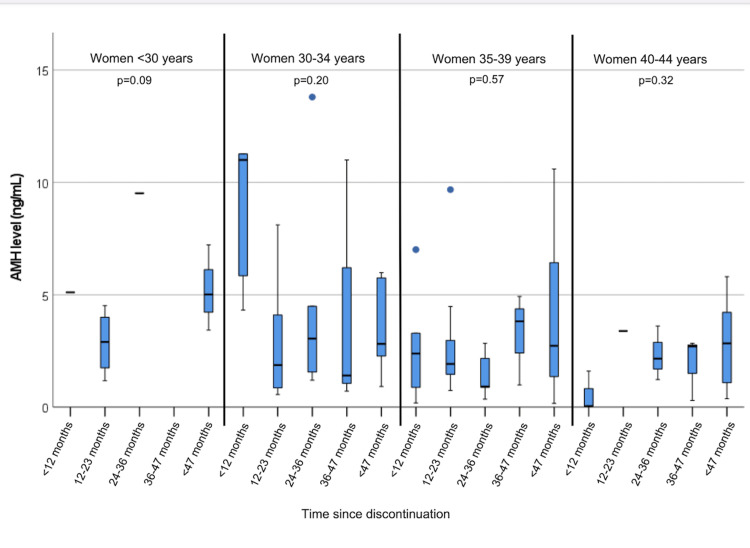
Median ovarian reserve levels as analyzed based on the time since the discontinuation of hormonal contraception use by age group (n=107) AMH: anti-Müllerian hormone

Among the 107 HC users (either short-term or long-term), the OR markers were not affected as analyzed based on the time since discontinuing the HC. However, age was a significant moderating factor in the relationship between time since discontinuing HC and AMH as well as AFC levels (Figure 2).

In bivariate analysis, time since discontinuing HC was marginally associated with median AMH levels for women aged less than 30 years (p=0.09). However, time since discontinuing HC was not associated with median AMH levels in any other age groups. In bivariate analysis, time since discontinuing HC was associated with cycle day three AFC levels among women aged 30-34 years (p=0.06), but not in any other age group. In linear regression analysis, women aged less than 30 years who had discontinued long-term HC between two and three years prior to their OR assessment had, on average, a 6.20 ng/mL increase in their AMH level compared to those who had discontinued HC less than two years prior to assessment (p=0.02) (Table 3).

**Table 3:** Simple linear regression results for the association between time since the discontinuation of HC use and ovarian reserve markers in specific age groups *p<0.05 ^1^Age groups included in regression models if p<0.1 in the Mann-Whitney Wilcoxon test. Multivariable regression models were not computed due to the small sample size in sub-groups AMH: anti-Müllerian hormone; AFC: antral follicle count; FSH: follicle-stimulating hormone; HC: hormonal contraception

Time since the discontinuation of HC use	AMH (women aged <30 years)^1^ n=9, β (95% CI)	AFC (women aged 30-34 years)^1^ n=38, β (95% CI)	AFC (women aged 40-44 years)^1^ n=19, β (95% CI)
<24 months	Ref.	Ref.	Ref.
24-47 months	6.20* (1.62 – 10.78)	-0.65 (-12.09 – 10.79)	2.75 (-9.89 – 15.39)
>47 months	1.9 (-1.15 – 4.95)	-0.68 (-11.15 – 9.78)	6.553 (-5.24 – 18.29)

The time since discontinuing long-term HC did not affect FSH or AFC levels, regardless of age in linear regression.

Use of artificial reproductive technology

Overall, 74 participants (60.2%) in the sample had used ART, with the most commonly used method being ovulation induction (n=53, 71.6%). Long-term HC users had a marginally though not significantly higher use of any ART compared to short-term/never users (64.3% vs. 44.0%, p=0.06). There were no differences between the long-term HC users and short-term/never HC users in the uptake of ovulation induction (71.4% vs. 72.7%, p=0.93), intrauterine insemination (IUI) (66.7% vs. 63.6%, p=0.85), or surrogacy (1.6% vs. 0, p=0.67); however, the long-term HC users did have a significantly higher rate of in vitro fertilization (IVF) use than short-term/never users (58.7% vs. 18.2%, p<0.01) (Table 1).

Outcomes of artificial reproductive technology

Of those who used ART (n=74), 32 participants (43.2%) reported a subsequent pregnancy. Short-term/never HC users had a higher rate of conception using any form of ART compared to long-term HC users (63.6% vs. 39.7%) but the difference was not statistically significant (p=0.14). Among those who used ovulation induction (n=53), short-term/never HC users had a significantly higher rate of conception compared to long-term HC users in bivariate analysis (75.0% vs. 15.6%, p<0.01). Among those who used IUI (n=49) also, short-term/never HC users had a significantly higher rate of conception compared to long-term HC users in bivariate analysis (57.1% vs. 21.4%, p=0.5). Pregnancy resulting from other forms of ART did not differ between long-term HC users and short-term/never users. In linear regression analysis, the odds of pregnancy from ART were not associated with a history of long-term HC use (Table 4).

**Table 4:** Simple and multivariable logistic regression results for variables associated with the odds of pregnancy and live birth among women using artificial reproductive technology (n=74) *p<0.05 ^1^Reference group: short-term/never HC users. ^2^All variables listed were included in the model. ^3^Could not converge due to a lack of variation in groups and too few participants. ^4^Surrogacy and Intra-fallopian transfer were not included due to too few participants (n=1 and n=0, respectively). ^5^Reference group: women who did not use this specific ART method OR: odds ratio; aOR: adjusted odds ratio; ART: assisted reproductive technology; HC: hormonal contraception; PCOS: polycystic ovarian syndrome; POI: primary ovarian insufficiency

Variable	Pregnancy with ART		Live birth with ART	
	Unadjusted model	Adjusted model^2^	Unadjusted model	Adjusted model^2^
	OR (95% CI)	aOR (95% CI)	OR (95% CI)	aOR (95% CI)
Long-term HC use^1^	0.37 (0.10 – 1.42)	0.38 (0.09 – 1.65)	0.26* (0.07 – 0.98)	0.30 (0.06 – 1.39)
Age, years	1.01 (0.90 – 1.13)	0.98 (0.86 – 1.11)	1.09 (0.95 – 1.25)	1.11 (0.96 – 1.27)
PCOS	0.79 (0.27 – 2.33)	0.61 (0.15 – 1.11)	1.91 (0.62 – 5.88)	1.86 (0.42 – 8.20)
POI	0.42 (0.04 – 4.27)	0.44 (0.04 – 4.69)	--^3^	--^3^
Type of ART used^4^				
Ovulation induction^5^	0.78 (0.28 – 2.15)	0.92 (0.29 – 2.96)	1.38 (0.43 – 4.42)	1.25 (0.29 – 5.30)
Intrauterine insemination^5^	1.22 (0.46 – 3.25)	1.29 (0.43 – 3.85)	1.02 (0.35 – 3.00)	0.93 (0.29 – 3.31)
In vitro fertilization^5^	1.03 (0.41 – 2.59)	1.58 (0.52 – 4.75)	0.75 (0.27 – 2.07)	1.23 (0.35 – 4.39)

In bivariate analysis, long-term HC users had lower rates of live birth overall after using ART compared to the short-term/never users (23.8% vs. 54.6%, p=0.04). In particular, among those who used ovulation induction (n=53), short-term/never HC users had a significantly higher rate of higher live birth rate compared to long-term HC users (62.5% vs. 8.9%, p<0.01). Live birth rates among those using other forms of ART did not differ between long-term HC users and short-term/never users. In the simple logistic regression, long-term HC users had 74% reduced odds of live birth resulting from ART compared to short-term/never users (p<0.05). However, after adjusting for covariates of age, diagnosis of PCOS or POI, and type of ART method, there were no significant differences in the odds of live birth between the long-term HC users and short-term/never users (aOR: 0.30, 95% CI: 0.06-1.39) (Table 4).

## Discussion

**Figure 3:**
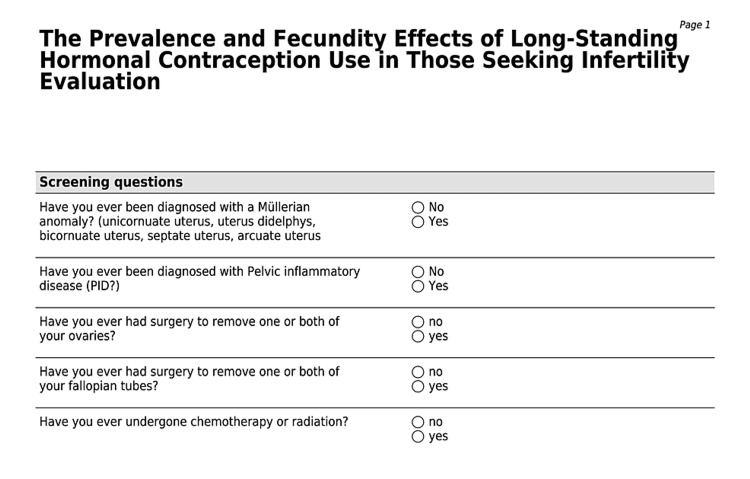
Study instrument

In this study, we found that OR markers including serum AMH, FSH, and AFC in long-term HC users (≥2 years) were overall similar to short-term (<2 years) or never users, though long-term HC users reported a higher rate of ART use, especially IVF. While the long-term HC users did have lower rates of conception with the use of ovulation induction and IUI, they did not have reduced odds of conception overall. Similarly, long-term HC users had lower rates of live birth resulting from ART, namely from ovulation induction, but after adjusting for age, diagnosis of PCOS or POI, and type of ART used, the odds of live-birth outcomes were not found to be affected by a history of long-term HC use. Through our patient survey (Figure 3) and retrospective medical chart review of 123 otherwise healthy women seeking an evaluation for unexplained infertility, we add to the growing body of literature highlighting the effects of HC use on various OR markers [12-15,20]. However, the findings from our study are novel in that our population was limited to those with primary infertility who had discontinued their HC on average 2.8 years prior to the evaluation. It is also the first study to date to report specifically on the comparison of uptake and outcomes of ART between long-term HC users and short-term/never users.

Although each hormonal contraception has a slightly different primary mechanism of action to prevent pregnancy, they all act either fully or partially to suppress gonadotropin secretion by the pituitary gland to arrest follicle growth, inhibit ovulation, and reduce hormone production. This interplay with the HPO axis has been shown to have consistent and intuitive inhibitory effects on FSH and thus AFC and/or ovarian volume [27]. With the exception of Deb et al., studies investigating the effects of current long-term HC use on AMH have similarly shown lower values than non-users, likely due to the decrease in granulosa cell stimulation of small antral follicles by the reduced levels of circulating FSH [8,13,14,28]. Nonetheless, while we found that long-term HC users had, on average, a 25% lower AMH, a 17% higher FSH, and a 31% lower AFC, these findings were not statistically significant even after adjusting for age and diagnosis of PCOS or POI in linear regression models. However, we did find that age played a role in the temporary suppressive effects of HC use on AMH levels. Women in our study who were aged less than 30 years had, on average, a 6.20 ng/mL increase in AMH levels after discontinuing HC for two to three years as compared to those who had discontinued HC use for less than two years prior to the evaluation. Other studies have also found that the AMH suppression from HC use is likely temporary and should be reversible after discontinuation; nonetheless, the exact timing of this “wash-out” period is still unknown [22,28].

While the reduction in OR markers observed in long-term HC users in our study may not have been statistically significant, we believe that it was clinically significant given the higher number of long-term users reporting subjective difficulties in becoming pregnant as well as the increased use of ART seen in long-term users despite otherwise comparable demographic characteristics and infertility risk factors. Specifically, 64.3% of long-term HC users reported utilizing some form of ART to achieve their goals of pregnancy compared to 44.0% of short-term/never users. Additionally, a significantly higher number of long-term users opted for the use of IVF. The authors hypothesize that the reason for the higher prevalence of utilizing this more aggressive treatment option among long-term HC users was twofold: (1) given the slightly lower AMH/AFC and higher FSH levels seen in long-term HC users, this treatment option may have been more readily recommended by the reproductive endocrinology and infertility (REI) provider, and (2) as highlighted by the lower success rates after ovulation induction treatment, long-term HC users may have a delayed return of a functioning HPO axis leading to less success with natural and ovulation induction cycles.

Nonetheless, findings from our study provide similar reassurance as previously published studies in that overall pregnancy and live-birth rates should not be affected by a history of long-term HC use [18,24,29]. Apart from a higher live-birth rate after ovulation induction among short-term/never users, our study found no differences in IUI or IVF outcomes in live birth outcomes overall after adjusting for age, diagnosis of PCOS or POI, and type of ART used.

Our findings are limited by certain factors. Firstly, the retrospective nature of the study inherently caused a lack of completely standardized data collection despite infertility work-up protocols implemented at CU ARM. While limiting our study population to those seen at an infertility clinic provides useful information on this group of patients, the findings are not generalizable to the general population, thereby limiting the external validity of our results. Additionally, the response rate in the current study to our questionnaire was 18.4% and hence may not be representative of our entire intended study population. The study population selection was also not powered to detect any racial or ethnic differences in our findings, either between any specific types or dosages of the HC. Furthermore, the patient survey used to obtain data regarding HC duration, type, and timing of cessation relied on subjective reporting and may have been susceptible to recall bias. Though the authors infer that some of the increased uptake of ART seen in long-term HC users may be attributed to the longer time required to conceive with natural conception, this information was not readily available. Lastly, despite the best attempts to limit confounding variables, the authors are aware that infertility and thus the use and success rates of various ART methods are multifactorial and complex topics. 

The overall strengths of this study include the novelty of the research question investigated as well as the fact that all patients had been treated at the same institution. Having similar specimen handling, laboratory evaluation techniques, ultrasonographer assessment, and infertility work-up protocols helps to control for subtle differences in OR markers, and its lack had been noted as a limitation in previously published meta-analyses comparing OR markers between various laboratories [30]. Additionally, we attempted to control for other confounding variables that could affect the prevalence of ART use and subsequent outcome(s) by using strict inclusion and exclusion criteria of participants, and similar baseline characteristics between the two groups also helped in this regard. Furthermore, our logistic regression analysis helped to control for patient age and diagnosis of PCOS or POI.

## Conclusions

Despite the above-mentioned limitations, the findings from this study highlight that the length of time since a patient has discontinued long-term HC may temporarily affect certain OR markers, especially if the patient is less than 30 years old. Our study also suggests that long-term HC users may employ an increased use of ART, specifically IVF. Overall, however, a history of long-term HC use should not impact live-birth outcomes; however, we recommend larger prospective studies to substantiate these findings. Nonetheless, results from this study can be used to counsel patients struggling with infertility and should be discussed with all women seeking regular preventive services.
